# The relationship between Helicobacter pylori infection and intestinal parasites in individuals from Khartoum state, Sudan: a case-control study

**DOI:** 10.12688/f1000research.21397.2

**Published:** 2021-01-18

**Authors:** Yasir Yousif Abd Elbagi, Ahmed Bakheet Abd Alla, Mohammed Baha Eldin Saad

**Affiliations:** 1 Department of Parasitology and Medical Entomology, College of Medical Laboratory Science, Sudan University of Science and Technology, Khartoum, Sudan; 2 Department of Parasitology and Medical Entomology, College of Medical Laboratory Science, Omdurman Ahlia University, Omdurman, Sudan

**Keywords:** H.pylori, Intestinal parsasite, Khartoum, Alsaha, Yastabsheron

## Abstract

**Background:** In developing countries, *Helicobacter pylori* infection is common, as are intestinal parasites. Socioeconomic circumstances and low personal hygiene lead to the spread of these infections. This research aimed to evaluate the relationship between intestinal parasites and *H. pylori *in Khartoum, Sudan.

**Methods:** This study was conducted in various hospitals in Khartoum between June and October 2018. The study involved 200 individuals: 100 patients with *H. pylori *as a case group and 100 healthy individuals as a control group. A stool sample was taken from each individual, and wet preparation, saturated sodium chloride flotation and formal ether concentration were used to detect intestinal parasites.

**Results:** The results showed that 23% of *H. pylori* patients and 10% of healthy individuals had gastrointestinal parasites; *Entamoeba histolytica *was found in 12% of *H. pylori* cases followed by *Entamoeba coli* (7%) and *Giardia lamblia* (4%). Control group: *Entamoeba histolytica* in 5% followed by *G. lamblia* in 3% and E. coli in 2% of individuals. There was a significant difference in the prevalence of intestinal parasites between groups (P = 0.013).

The prevalence rate of intestinal parasites among men and women was 24% and 22%, respectively, in the case group, and 9% and 11%, respectively, in the control group. In the case group, the highest prevalence rates (40% and 38%) were found among the age groups 1-15 and 46-60 years old, respectively, while the lowest rate (10.7%) was found among the 31-45 age group. In the control group, the highest prevalence rate (15%) was among the 31-45 age group, the lowest prevalence rate (8%) was found among the 16-30 age group.

**Conclusion: **Together, we found that intestinal parasites are more common in patients with *H. pylori*. We also noticed that the rate of infection was not affected by gender while the age group was affected.

## Introduction

Among the most common diseases in the world are intestinal parasite infections; an estimated 3.5 billion people are affected, and 450 million people are infected ( Jayalakshmi & Dharanidevi, 2016). These infections are considered a serious public health problem as they cause anaemia with iron deficiency, retardation of growth in children, and other physical and mental health problems ( Okyay *et al.*, 2004; Tandukar *et al.*, 2013; Wongstitwilairoong *et al.*, 2007). One example is a pathogenic intestinal protozoon that infects the small and/or large intestine ( Farthing & Kelly, 2005), or an intestinal worm, such as *Ascaris lumbricoides*, *Trichuris trichiura*, *Enterobius vermicularis,* and hookworms, which affect people in tropical countries ( Smyth, 1990).

Health impacts differ with age: the small intestinal protozoa *Giardia lamblia* and *Cryptosporidium* spp. have a serious impact on children ( Harhay *et al.*, 2010), while the large intestine pathogen *Entamoeba histolytica* has a higher morbidity among adults of all ages ( Mortimer & Chadee, 2010). Some protozoa, in particular, *Cryptosporidium* and *Isospora belli*, cause significant morbidity in individuals with immunodeficiency ( Bachur *et al.*, 2008), for example giardiasis and amoebiasis are opportunistic parasites ( Biggs & Brown, 2001). *Helicobacter pylori* is the most common chronic human bacterial infection, infecting 70–90% of the population of developing countries and 25–50% of the people of developed countries ( Gillespie & Hawkey, 2006). *H. pylori* colonizes the stomach’s mucus layer and induces chronic active gastritis inflammation ( Konturek, 2003). It is a major cause of peptic ulcers and a risk factor for gastric malignancies ( Lesbros-Pantoflickova *et al.*, 2007).

*H. pylori* can be easily identified in all microbiology laboratories using simple techniques ( Guerrant *et al.*, 2011). Numerous serological diagnostic tests used for the detection of *H. pylori* include bacterial agglutination, complement fixation, indirect immunofluorescence test, enzyme immunoassay, and enzyme-linked immunosorbent assay ( Kim, 2016). Since *H. pylori* and intestinal parasites are prevalent in developing countries, this study aimed to determine the prevalence of intestinal parasites in patients with *H. pylori* in Khartoum state.

## Methods

### Study design

This was a case-control study carried out in Khartoum state, Sudan, at Alsaaha Specialized Hospital and Yastabshiron Hospital between 1 June and 27 October 2018. The study was conducted in 100 patients who were *H. pylori* positive as a case group and 100 individuals without *H. pylori* as a control group. The *H. pylori* test had already been performed in hospitals using immuno-chromatographic test (ICT) for identification of *H. pylori* Ag in stool sample. Participants were divided into groups according to gender and age (see below).

### Ethical considerations

Approval for the study was obtained from the Ethical Authorization Committee (number, MLS-IEC-10-17) of the College of Medical Laboratory Sciences, Sudan University of Science and Technology. Written informed consent for participation and disclosure of data was obtained from each participant in the study and in the case of children (<18 years) written informed consent was obtained from their guardians.

### Participants

Individuals in this study who were already being screened for presence of *H. pylori* using ICT for antigen in stool were asked to participate. Individuals with a positive ICT for *H. pylori* antigen were included in the case group after they agreed to participate in the study, while the control group were those with negative *H. pylori* antigen, who were also only included in the study after they agreed to participate.

### Sampling collection

In total, 200 stool samples were collected from the participants in the study. Samples were collected immediately after participants agreed to partake in the study. Each participant was provided with a labelled stool container (transparent and clean) and was instructed to collect a faecal sample.

### Method of stool examination

Every stool sample was examined for the detection of the intestinal parasite by wet preparation, saturated sodium chloride floatation and formal ether concentration. If one detection method was positive, then the sample was counted as positive for intestinal parasites, even if other methods were negative.

***Wet preparation***. A small portion of stool was mixed with a drop of normal saline with a wooden applicator stick and deposited on a slide. This was covered with a cover slip and routinely examined under a microscope using 10X and a high magnification 40X to detect more parasites, as per the World Health Organization protocol ( WHO, 2001).

***Formal ether concentration***. Approximately 1 g of faeces from various parts of the stool was collected and emulsified in a glass beaker in 5 ml of formal saline. There a further 5 ml of saline was added and mixed. The resulting suspension was strained using a sieve with small pores. The filtered sample was poured into a centrifugal tube, and an equal volume of ether was added. For one minute, the tube was mixed and then centrifuged at 2000 rpm for 5 minutes. The upper three layers were discarded, and the sediment was moved to a slide, covered with a cover slip and analysed under a microscope using magnifications 10X and 40X. This was as per the protocol by Smith & Mank (2011).

***Saturated sodium chloride floatation***. Approximately a 0.5 g of faeces was collected from different parts of the stool and emulsified in a long glass tube half-filled with saturated sodium chloride solution. Then the container was filled with sodium chloride until the top of tube. Carefully, a cover glass was put on the top of the tube avoiding air bubbles. After 30 to 45 minutes, the cover glass was removed from the top of the tube and put on a clean and dry slide and examined under the microscope using 10X and 40X magnifications. This was as per the protocol by Dryden *et al.* (2005).

### Data analysis

Statistical analysis was performed using SPSS version 20.0. The Chi-square method was used to compare variables. P values < 0.05 were considered statistically significant.

## Results

The results showed that 23 of the 100 patients with *H. pylori* were infected with gastrointestinal parasites (23%). Of the 100 control individuals, 10 were found to be infected with gastrointestinal parasites (10%). Between the case and control groups, there was a statistically significant difference in prevalence of intestinal parasites ( *P* = 0.013).

Among *H. pylori* patients, the occurrence of intestinal parasites in men and women was similar (24% and 22%, respectively; *P* = 0.841; Table 1). On the other hand, the prevalence of gastrointestinal parasites in men and women in the control group was found to be 9% and 11%, respectively, but this difference was not statistically significant ( *P* = 0.789; Table 1).

**Table 1.** Occurrence of gastrointestinal parasites among *H. pylori* patients and control individuals according to gender.

Gender	N examined		N positive (%)	
	*Case*	*Control*	*Case*	*Control*
Male	46	54	11 (24)	5 (9)
Female	54	46	12 (22)	5 (11)
Total	100	100	23 (23)	10 (10)

Case difference between groups: *P* = 0.841; Control difference between groups: P = 0.789.

In the case group, the highest occurrence rates (40% and 38%) were reported among the 1–15 and 46–60 age groups, while the lowest rate (10.7%) was reported among the 31–45 age group. These differences were not statistically significant ( *P*. value= 0.132; Table 2). For the control group, the highest prevalence rate (15%) was reported among the 31–45 age group, while the lowest prevalence rate was among the 16–30 age group (8%). This difference was not statistically significant (P = 0.528; Table 2).

**Table 2.** Occurrence of gastrointestinal parasites among *H. pylor* *i* patients and control individuals according age group.

Age group, years	N examined		N positive (%)	
	*Case*	*Control*	*Case*	*Control*
1–15	10	14	4 (40)	2 (14.3)
16–30	37	37	7 (19)	3 (8)
31–45	28	33	3 (10.7)	5 (15)
46–60	21	12	8 (38)	0 (0)
61–75	4	4	1 (25)	0 (0)
Total	100	100	23 (23)	10

Case difference between age groups: *P =* 0.132; Control difference between age groups: P = 0.528.

The results showed that *Entamoeba histolytica* was seen in 12% of *H. pylori* cases followed by *Entamoeba coli* in 7% and *G. lamblia* in 4% of cases ( Table 3). Among the control group *E. histolytica* was reported at 5%, followed by *G. lamblia* at 3% and *E. coli* at 2% ( Table 3).

**Table 3.** Occurrence of different gastrointestinal parasites in *H. pylor* *i* patients and control individuals.

Parasite	N examined		N positive (%)	
	*Case*	*Control*	*Case*	*Control*
*Entamoeba histolytica *	100	100	12 (12)	5 (5)
*Giardia lamblia *	100	100	4 (4)	3 (3)
*Entamoeba coli *	100	100	7 (7)	2 (2)

## Discussion

From the study, it is evident that the gastrointestinal parasite overall occurrence among *H. pylori* patients is relatively high (23%). It was found that this rate was higher than the published rate by Uğraş & Miman (2014) in Turkey (7.61%). As far as the control group is concerned, the overall occurrence rate reported was 10%. This rate is lower than the rate among *H. pylori* patients and higher than the rate reported by Uğraş & Miman (2014). The difference in rates between the control group and patient with *H. pylori* was significant. This, in our opinion, might mean that there is an association between the establishment of gastrointestinal parasites and *H. pylori*.

The difference in prevalence rates between men and women in *H. pylori* patients and control individuals was not statistical difference. This finding did not agree with Yakoob *et al.* (2005), who considered *G. lamblia* occurrence in Pakistan. That study found a higher rate in men (72%) than in women (28%).

In our study, the highest occurrences (40% and 38%) were reported among the 1–15 and 46–60 year age groups, respectively, in the *H. pylori* patients, and the 31–45 year age group (15%) for the control group. Our finding disagreed with the finding of Fadul *et al.* (2016), who reported the highest occurrence rate (50%) in the age group >66 years old. Our results also showed that *E. histolytica* was seen in 12% of the *H. pylori* cases followed by *E. coli* in 7% of cases and *G. lamblia* in 4%. Lower rates were reported among the control group where *E. histolytica* was seen in 5% followed by *G. lamblia* in 3% and *E. coli* in 2%. Our result are not in line with the findings of Gökşen *et al.* (2016) who reported 14.8% for *G. lamblia* in the *H. pylori*-positive group, which was in agreement with Escobar-Pardo *et al.* (2011) who also found a significant association between *H. pylori* and *G. lamblia*. However, our conclusion was in total disagreement with the finding of Seid *et al*. (2018) in Ethiopia, who reported no significant association between *H. pylori* and *E.histolytica* but there was significant association with *G.lamblia*. This may be due to differences in the study areas.

## Conclusions

Gastrointestinal parasites are more common among *H. pylori* patients compared to individuals without *H. pylori*; but this infection rate was not affected by gender. The highest infection rate was reported in the 1–15 and 46–60 age group among *H. pylori* patients and 31–45 years of age group among the control patients.

## Data availability

### Underlying data

Figshare: yasir and ahmed.sav, https://doi.org/10.6084/m9.figshare.10315769.v2 ( Abd Alla & Yousif, 2019).

This project contains the following underlying data:

- Raw data file.sav
- Data dictionary

Data are available under the terms of the Creative Commons Zero “No rights reserved” data waiver (CC0 1.0 Public domain dedication).

## References

[ref-1] Abd Alla A Yousif Y: yasir and ahmed.sav. *figshare.*Dataset.2019 10.6084/m9.figshare.10315769.v2

[ref-2] Bachur TP Vale JM Coêlho IC: Enteric parasitic infections in HIV/AIDS patients before and after the highly active antiretroviral therapy. *Braz J Infect Dis.* 2008;12(2):115–122. 10.1590/s1413-86702008000200004 18641847

[ref-3] Biggs B Brown G: Principles and Practice of Clinical Parasitology. *Principles and Practice of Clinical Parasitology.*Edited by Stephen H.gillespie and R. D. Pearson.2001 10.1002/0470842504.ch3

[ref-4] Dryden MW Payne PA Ridley R: Comparison of common fecal flotation techniques for the recovery of parasite eggs and oocysts. *Vet Ther.* 2005;6(1):15–28. 15906267

[ref-5] Escobar-Pardo ML de Godoy AP Machado RS: Prevalence of *Helicobacter pylori* infection and intestinal parasitosis in children of the Xingu Indian Reservation. *J Pediatr (Rio J).* 2011;87(5):393–8. 10.2223/JPED.2118 21842116

[ref-6] Fadul N Ahmed M Elamin T: Prevalence Rate Of *Giardia Lamblia* / *Helicobacter pylori* Co-Infections In Khartoum State, Sudan. *International Journal of Scientific & Technology Research.* 2016;5(3):181–190. Reference Source

[ref-7] Farthing MJ Kelly P: Protozoal gastrointestinal infections. *Medicine.* 2005;33(4):81–83. 10.1383/medc.33.4.81.64360

[ref-8] Gillespie SH Hawkey PM: Principles and Practice of Clinical Bacteriology Second Edition. Principles and Practice of Clinical Bacteriology.2006 10.1002/9780470017968

[ref-9] Gökşen B Appak YÇ Girginkardesler N: Coexistence of *Helicobacter pylori* and Intestinal Parasitosis in Children with Chronic Abdominal Pain. *Turkiye Parazitol Derg.* 2016;40(1):32–6. 10.5152/tpd.2016.4508 27222333

[ref-10] Guerrant RL Walker DH Weller PF: Tropical infectious diseases: principles, pathogens and practice.third edit. Elsevier Inc.2011 10.1016/C2009-0-40410-0

[ref-11] Harhay MO Horton J Olliaro PL: Epidemiology and control of human gastrointestinal parasites in children. *Expert Rev Anti Infect Ther.* 2010;8(2):219–234. 10.1586/eri.09.119 20109051 PMC2851163

[ref-12] Jayalakshmi S Dharanidevi S: The Prevalence of Intestinal Parasitic Infections in a tertiary care hospital in southern India-A retrospective study. *Int J Curr Microbiol App Sci.* 2016;5(10):718–23. 10.20546/ijcmas.2016.510.078

[ref-13] Kim N: *Helicobacter pylori*.Edited by N. Kim. Springer Nature.2016 10.1007/978-981-287-706-2

[ref-14] Konturek JW: Discovery by Jaworski of *Helicobacter pylori* and its pathogenetic role in peptic ulcer, gastritis and gastric cancer. *J Physiol Pharmacol.* 2003;54 Suppl 3:23–41. 15075463

[ref-15] Lesbros-Pantoflickova D Corthésy-Theulaz I Blum AL: *Helicobacter pylori* and probiotics. *J Nutr.* 2007;137(3 Suppl 2):812S–8S. 10.1093/jn/137.3.812S 17311980

[ref-16] Mortimer L Chadee K: The immunopathogenesis of *Entamoeba histolytica*. *Exp Parasitol.* 2010;126(3):366–380. 10.1016/j.exppara.2010.03.005 20303955

[ref-17] Okyay P Ertug S Gultekin B: Intestinal parasites prevalence and related factors in school children, a western city sample--Turkey. *BMC Public Health.* 2004;4:64. 10.1186/1471-2458-4-64 15615592 PMC544355

[ref-21] Seid A Tamir Z Kasanew B: Co-infection of intestinal parasites and *helicobacter pylori* among upper gastrointestinal symptomatic adult patients attending Mekanesalem hospital, Northeast Ethiopia. *BMC Res Notes.* 2018;11(1):144. 10.1186/s13104-018-3246-4 29463293 PMC5819640

[ref-18] Smith HV Mank TG: Diagnosis of human giardiasis.In *Giardia* Springer, Vienna.2011;353–377. 10.1007/978-3-7091-0198-8_22

[ref-19] Smyth JD: Foundations of parasitology (4th edn).8th edn, *Parasitol Today* 8th edn. Edited by P. E. Reidy. Americas, New York: Janice Roerig-Blong.1990 10.1016/0169-4758(90)90219-T

[ref-20] Tandukar S Ansari S Adhikari N: Intestinal parasitosis in school children of Lalitpur district of Nepal. *BMC Res Notes.* 2013;6(1):449. 10.1186/1756-0500-6-449 24207086 PMC3829703

[ref-22] Wongstitwilairoong B Srijan A Serichantalergs O: Intestinal parasitic infections among pre-school children in Sangkhlaburi, Thailand. *Am J Trop Med Hyg.* 2007;76(2):345–350. 10.4269/ajtmh.2007.76.345 17297047

[ref-25] Uğraş M Miman O: The prevalence of intestinal parasites in children with *Helicobacter pylori* gastritis evaluated retrospectively. *Turkiye Parazitol Derg.* 2014;37(4):245–248. 10.5152/tpd.2013.3191 24412863

[ref-23] World Health Organization: Guidelines on standard operating procedures for laboratory diagnosis of HIV-oportunistic infections (No. SEA-HLM-332). WHO Regional Office for South-East Asia.2001 Reference Source

[ref-24] Yakoob J Jafri W Abid S: Giardiasis in patients with dyspeptic symptoms. *World J Gastroentrol.* 2005;11(42):6667–6670. 10.3748/wjg.v11.i42.6667 16425362 PMC4355762

