# Supplementary material for: The relationship between Helicobacter pylori infection and intestinal parasites in individuals from Khartoum state, Sudan: a case-control study
Source: F1000Res. 2021 Jan 18;8:2094. Originally published 2019 Dec 12. [Version 2] doi: 10.12688/f1000research.21397.2 (PMC7385544; doi:10.12688/f1000research.21397.2)
Supplement: Peer review history

# Peer review: Reviewer response for version 2

**DOI:** 10.5256/f1000research.47350.r77618

**Published:** 2021-01-19

**Authors:** Paweł Krzyżek

**Affiliations:** 1 Department of Microbiology, Wroclaw Medical University, Wroclaw, Poland

The quality of the article has improved. I still think that in the discussion it would be beneficial to include the part about the influence of the host immune system on the observed results (which was not included), but I leave it to the Authors' choice.

Is the work clearly and accurately presented and does it cite the current literature?

Partly

If applicable, is the statistical analysis and its interpretation appropriate?

Partly

Are all the source data underlying the results available to ensure full reproducibility?

Yes

Is the study design appropriate and is the work technically sound?

Yes

Are the conclusions drawn adequately supported by the results?

Yes

Are sufficient details of methods and analysis provided to allow replication by others?

Yes

Reviewer Expertise:

Microbiology, mostly focused on H. pylori

I confirm that I have read this submission and believe that I have an appropriate level of expertise to confirm that it is of an acceptable scientific standard.

# Peer review: Reviewer response for version 2

**DOI:** 10.5256/f1000research.47350.r77620

**Published:** 2021-01-19

**Authors:** Elena Pomari

**Affiliations:** 1 Department of Infectious-Tropical Diseases and Microbiology, IRCCS Sacro Cuore Don Calabria Hospital, Verona, Italy

No further comments to make.

Is the work clearly and accurately presented and does it cite the current literature?

Partly

If applicable, is the statistical analysis and its interpretation appropriate?

Yes

Are all the source data underlying the results available to ensure full reproducibility?

Yes

Is the study design appropriate and is the work technically sound?

Yes

Are the conclusions drawn adequately supported by the results?

Partly

Are sufficient details of methods and analysis provided to allow replication by others?

Yes

Reviewer Expertise:

Infectious diseases

I confirm that I have read this submission and believe that I have an appropriate level of expertise to confirm that it is of an acceptable scientific standard.

# Peer review: Reviewer response for version 1

**DOI:** 10.5256/f1000research.23567.r67155

**Published:** 2020-08-10

**Authors:** Yeong Yeh Lee

**Affiliations:** 1 Department of Medicine, School of Medical Sciences, Universiti Sains Malaysia, Kota Bharu, Malaysia

The study adds to a body of literature supporting the common presence of *H. pylori* and intestinal parasites co-infection. There are several concerns as below:

1. Best if the study design involved age and gender-matched cohorts. Case-control might be biased especially selection bias.
2. Sample size calculation - how 100 samples each arm is decided?
3. Ideally, two serological tests for *H. pylori *are needed to confirm infection.
4. Only limited parasites were detected. Please provide explanations. 
5. Please discuss reasons for co-infection of detected parasites with *H. pylori*, the significance, and discuss the age group differences found between current study and others.

Is the work clearly and accurately presented and does it cite the current literature?

Partly

If applicable, is the statistical analysis and its interpretation appropriate?

Partly

Are all the source data underlying the results available to ensure full reproducibility?

Yes

Is the study design appropriate and is the work technically sound?

Partly

Are the conclusions drawn adequately supported by the results?

Partly

Are sufficient details of methods and analysis provided to allow replication by others?

Yes

Reviewer Expertise:

Helicobacter pylori, gut microbiota, gastrointestinal cancers

I confirm that I have read this submission and believe that I have an appropriate level of expertise to confirm that it is of an acceptable scientific standard, however I have significant reservations, as outlined above.

## References

[1] : Dual infective burden of Helicobacter pylori and intestinal parasites: Good or bad news for the host?. *Indian Journal of Gastroenterology*.2020;39(2) : 10.1007/s12664-020-01045-8 111-116 10.1007/s12664-020-01045-8 32372188

# Peer review: Reviewer response for version 1

**DOI:** 10.5256/f1000research.23567.r67158

**Published:** 2020-07-27

**Authors:** Asmaa Ibrahim

**Affiliations:** 1 Department of Molecular biology, Genetic Engineering and Biotechnology Research Institute, University of Sadat city, Sadat, Egypt

The article aims to evaluate the prevalence rate of intestinal parasites in *H. pylori *positive patients in a specific state in Sudan.

**Major revision** **:**

1. The introduction needs to be more updated (ex. Haque (2007) and Nyantekyi et al., (2010) instead of (Smyth, 1990), both articles associated with helminthic infections in developing countries.
2. In the method of stool examination part, I prefer to make also examination with acid-fast stain (AF), in case of diarrhea or immunodeficiency to detect coccidian parasites.
3. In data analysis part, if there are any data about socio-demographic characteristics such as (residency in rural or urban areas, animal contact, and water source) and clinical symptoms such as (diarrhea, vomiting and fever) were collected, I prefer to analyze its association with the infection as estimated risk factors (multivariate logistic regression, 95% CI and OR).
4. The current study associated with the " developing countries" especially Sudan, while the discussion is focused on studies conducted a specific country “Turkey”, which mainly not considered as a developing country. I prefer to compare the present study with other studies in Egypt (10.1007/s12639-018-1075-y ^1^), Iran (10.5812/pedinfect.15294 ^2^) and Ethiopia (10.1186/s13104-018-3246-4 ^3^). These countries more suitable for that topic.

Is the work clearly and accurately presented and does it cite the current literature?

Partly

If applicable, is the statistical analysis and its interpretation appropriate?

Partly

Are all the source data underlying the results available to ensure full reproducibility?

Yes

Is the study design appropriate and is the work technically sound?

Yes

Are the conclusions drawn adequately supported by the results?

Yes

Are sufficient details of methods and analysis provided to allow replication by others?

Yes

Reviewer Expertise:

Infectious diseases, mostly focused on H. pylori and Cryptosporidium

I confirm that I have read this submission and believe that I have an appropriate level of expertise to confirm that it is of an acceptable scientific standard, however I have significant reservations, as outlined above.

## References

[1] : Helicobacter pylori and enteric parasites co-infection among diarrheic and non-diarrheic Egyptian children: seasonality, estimated risks, and predictive factors. *J Parasit Dis*.2019;43(2) : 10.1007/s12639-018-1075-y 198-208 10.1007/s12639-018-1075-y 31263324PMC6570731

[2] : Estimation of the Parasitic Infection Prevalence in Children With Helicobacter pylori Infection in Ilam City (2012-2013). *Archives of Pediatric Infectious Diseases*.2014;2(1) : 10.5812/pedinfect.15294 10.5812/pedinfect.15294

[3] : Co-infection of intestinal parasites and Helicobacter pylori among upper gastrointestinal symptomatic adult patients attending Mekanesalem Hospital, northeast Ethiopia. *BMC Res Notes*.2018;11(1) : 10.1186/s13104-018-3246-4 144 10.1186/s13104-018-3246-4 29463293PMC5819640

# Peer review: Reviewer response for version 1

**DOI:** 10.5256/f1000research.23567.r67152

**Published:** 2020-07-21

**Authors:** Paweł Krzyżek

**Affiliations:** 1 Department of Microbiology, Wroclaw Medical University, Wroclaw, Poland

The article is a short report which aims to show the higher frequency of selected parasites in *H. pylori*-positive patients. I believe the manuscript is worth indexing, however, I suggest to apply two corrections:

- I think that it would be easier for readers to give p-values in tables as an independent column (this way it will be easy to read which results are statistically different from each other).
- I believe that the discussion should be extended to the immunological part, even though it was not the aim of this article. The results obtained by the Authors indicate a higher frequency of parasites in *H. pylori*-positive patients aged 1-15 and 46-60. These results indicate that an immature/weakened immune system may influence the parasite- *H. pylori* relationship in the host. Thus, I suggest that the Authors should discuss this topic and add relevant literature references.

Is the work clearly and accurately presented and does it cite the current literature?

Partly

If applicable, is the statistical analysis and its interpretation appropriate?

Partly

Are all the source data underlying the results available to ensure full reproducibility?

Yes

Is the study design appropriate and is the work technically sound?

Yes

Are the conclusions drawn adequately supported by the results?

Yes

Are sufficient details of methods and analysis provided to allow replication by others?

Yes

Reviewer Expertise:

Microbiology, mostly focused on H. pylori

I confirm that I have read this submission and believe that I have an appropriate level of expertise to confirm that it is of an acceptable scientific standard.

# Peer review: Reviewer response for version 1

**DOI:** 10.5256/f1000research.23567.r67157

**Published:** 2020-07-15

**Authors:** Elena Pomari

**Affiliations:** 1 Department of Infectious-Tropical Diseases and Microbiology, IRCCS Sacro Cuore Don Calabria Hospital, Verona, Italy

Authors describe the occurrence of *H. pylori* and intestinal parasites concomitant infections in individuals from a specific state in Sudan.

**Major revision:**

1. .The rationale of the study is focused on the "in developing countries" and the discussion is mostly on studies conducted in Turkey. To the best of my knowlegde, the current (or recent) Turkey GDP is not so low. Thus, authors should compare their findings with other available data reported in studies conducted for example in Egypt, Ethiopia ... (doi: 10.12816/0010855 ^1^; 10.1186/s13104-018-3246-4 ^2^, etc).
2. In the discussion, authors conclude suggesting that different food and life style of individuals among different geo regions might determine various occurrence of infections. Did authors have any information about diet, life style of the included subjects? These variables could be added in tables and analysis.

**Minor revision:**

1. Keywords: check typing for "Intestinal parsaite" and "Yastabsheron"

Is the work clearly and accurately presented and does it cite the current literature?

Partly

If applicable, is the statistical analysis and its interpretation appropriate?

Yes

Are all the source data underlying the results available to ensure full reproducibility?

Yes

Is the study design appropriate and is the work technically sound?

Yes

Are the conclusions drawn adequately supported by the results?

Partly

Are sufficient details of methods and analysis provided to allow replication by others?

Yes

Reviewer Expertise:

Infectious diseases

I confirm that I have read this submission and believe that I have an appropriate level of expertise to confirm that it is of an acceptable scientific standard, however I have significant reservations, as outlined above.

## References

[1] : Prevalence of Helicobacter pylori infection among adult patients with different gastrointestinal parasites in Tanta City district. *J Egypt Soc Parasitol*.2015;45(1) : 10.12816/0010855 101-6 10.12816/0010855 26012224

[2] : Co-infection of intestinal parasites and Helicobacter pylori among upper gastrointestinal symptomatic adult patients attending Mekanesalem Hospital, northeast Ethiopia. *BMC Res Notes*.2018;11(1) : 10.1186/s13104-018-3246-4 144 10.1186/s13104-018-3246-4 29463293PMC5819640
